# Health-Related Coping and Social Interaction in People with Multiple Sclerosis Supported by a Social Network: Pilot Study With a New Methodological Approach

**DOI:** 10.2196/ijmr.7402

**Published:** 2017-07-14

**Authors:** Luigi Lavorgna, Antonio Russo, Manuela De Stefano, Roberta Lanzillo, Sabrina Esposito, Fatemeh Moshtari, Francesco Rullani, Kyrie Piscopo, Daniela Buonanno, Vincenzo Brescia Morra, Antonio Gallo, Gioacchino Tedeschi, Simona Bonavita

**Affiliations:** ^1^ University of Campania Luigi Vanvitelli 1st Clinic of Neurology Naples Italy; ^2^ University Federico II Department of Neurosciences, Reproductive Sciences and Odontostomatology Naples Italy; ^3^ Free International University for Social Studies Guido Carli Department of Business and Management Rome Italy

**Keywords:** social media, eHealth, digital health, multiple sclerosis, social network, Web medicine

## Abstract

**Background:**

Social media are a vital link for people with health concerns who find in Web communities a valid and comforting source for information exchange, debate, and knowledge enrichment. This aspect is important for people affected by chronic diseases like multiple sclerosis (MS), who are very well informed about the disease but are vulnerable to hopes of being cured or saved by therapies whose efficacy is not always scientifically proven. To improve health-related coping and social interaction for people with MS, we created an MS social network (SMsocialnetwork.com) with a medical team constantly online to intervene promptly when false or inappropriate medical information are shared.

**Objective:**

The goal of this study was to assess the impact of SMsocialnetwork.com on the health-related coping and social interaction of people with MS by analyzing areas of interest through a Web-based survey.

**Methods:**

Referring to previous marketing studies analyzing the online platform’s role in targeted health care, we conducted a 39-item Web-based survey. We then performed a construct validation procedure using a factorial analysis, gathering together like items of the survey related to different areas of interest such as utility, proximity, sharing, interaction, solving uncertainty, suggestion attitude, and exploration.

**Results:**

We collected 130 Web-based surveys. The areas of interest analysis demonstrated that the users positively evaluated SMsocialnetwork.com to obtain information, approach and solve problems, and to make decisions (utility: median 4.2); improve feeling of closeness (proximity: median 5); catalyze relationships and text general personal opinions (sharing: median 5.6); get in touch with other users to receive innovative, effective, and practical solutions (interaction, solving uncertainty, and suggestion attitude medians were respectively: 4.1, 3, and 3); and share information about innovative therapeutic approaches and treatment options (suggestion attitude: median: 3.3).

**Conclusions:**

SMsocialnetwork.com was perceived by users to be a useful tool to support health-related coping and social interaction, and may suggest a new kind of therapeutic alliance between physicians and people with MS.

## Introduction

Social media represent the most important virtual meeting places where users can get in touch with others, overcoming limitations of space and time [1,2] and may be considered a comforting space to share opinions and debate on disease-related concerns [3,4,5], especially for young people suffering from chronic diseases such as multiple sclerosis (MS) [6].

However, people with MS risk being exposed to inaccurate information on the Web [7] due to the assertive value of so-called patient-authored texts [8], which may be lacking in scientific and medically relevant evidence. To overcome this issue, in 2012 we created SMsocialnetwork.com, a social network to improve health-related coping and social interaction for people with MS **.**

The theoretical framework of this study has been suggested by the marketing approach proposed by Koh and Kim [9] in the work “Sense of Virtual Community,” implemented with both the theoretical model of Lazarus and Folkman [10], on psychological well-being during serious illness, and the transactional model of stress and coping [11].

We considered previous marketing studies to investigate SMsocialnetwork.com as a shoppable condition [12,13] (ie, a safe virtual space where one can find the sought-after health information [14-22]), evaluating the role of the online platform in targeted health care according to the clinical questions recommendations of evidence-based medicine (PICO: Problem/population, Intervention, Comparison, and Outcome) [23].

Our methodological approach assessed SMsocialnetwork.com using a Web-based survey in terms of the following areas of interest: utility (equivalent, in marketing research, to information need fulfillment [24]), proximity (measures and sources use of virtual co-presence [25]), sharing (use of self-presentation [23]), interaction (interpersonal communications [26]), solving uncertainty (perceived effectiveness [27]), suggestion attitude (knowledge contribution [26,28]), and exploration (personal innovativeness [29]). Analyzing items in the survey related to each of the 7 areas of interests, we performed a construct validation procedure. In other medical conditions, such HIV and heart disease, health-related Internet usage was associated with disease knowledge, information-seeking, coping, and using social supports as a stress buffer [30,31].

The aim of this study was to evaluate the impact of SMsocialnetwork.com on health-related coping and social interaction in people with MS by analyzing the above mentioned areas of interest through a Web-based survey.

## Methods

### SMsocialnetwork.com

SMsocialnetwork.com www.SMsocialnetwork.com is a Facebook-like social network dedicated to people with MS, created in 2012 by a group of neurologists and psychologists from the 1st Clinic of Neurology of the University of Campania “Luigi Vanvitelli” with vast experience in MS. It is based on Wordpress [32] and BuddyPress [33], two open source platforms for online communities. Several plug-ins are used to enhance and protect the online user experience.

In order to ensure proper use of the social network, we have guaranteed the constant online presence of neurologists amd psychologists from the medical team to oversee and participate on the public wall, intervening promptly in case of posts with false or inappropriate medical information. Neurologists and psychologists are not involved in technical aspects of the social network (graphic design, Web development, hosting services, and chat implementation were managed by the Web designer) but oversee the public activities of the users, post relevant information about MS, protect users from false rumors and fake news, answer questions via private or public message, and preserve users’ right to hope, in total respect of scientific rigor. Their intervention does not include banning users but does include explaining why that specific post is not scientifically correct. The SMsocialnetwork.com plug-ins were chosen in order to protect user privacy; at the subscription step, users had to agree with the privacy policy. Nonregistered users are able to view only the Welcome and About Us pages. Public wall, surveys, chat, and all other sections of the social network are restricted to registered users only.

SMsocialnetwork.com includes the following sections: (1) public wall and public posts, where users may read, write, post, comment, and get in touch with other users, which is continuously monitored by neurologists and psychologists; (2) streaming pages (oral communications at congresses, examples of outpatients visits, etc); (3) groups and forums on specific MS-related areas (pregnancy, pediatric demyelinating diseases, headache, sport, diet, etc); (4) links to scientific news or MS-related events; (5) private one-to-one and multiple chats; and (6) videos on specific topics uploaded by physicians on the SMsocialnetwork.com staff or external consultants with specific competence in MS. At the time of the investigation, SMsocialnetwork.com included 1020 active users (users who visited SMsocialnetwork.com and logged in over the 2 months prior to the study). The total number of pages viewed was 187,073, the average number of pages viewed per visit was 5, and the average duration of a session was 7 minutes 28 seconds.

### Standard Protocol Approvals, Registrations, and Patient Consents

The study was performed in accordance with good clinical practice and the Declaration of Helsinki. All participants consented to the use of recorded surveys for scientific purposes on aggregate level. To protect the anonymity of the participants, the Internet protocol codes of the computers were not registered and no electronic cookies were embedded.

### Web-Based Survey

We conducted a Web-based survey availing ourselves of a marketing approach generally used to learn how the system design and the social aspects of Web communities jointly influence members’ behavior and participation [24,25,34-36]. We used a 39-item survey posted from April to June 2015 on the SMsocialnetwork.com public wall (displayed in a pop-up window when visitors accessed the website).

The survey was created with the collaboration of the Department of Business and Management of the Free International University for Social Studies “Guido Carli” (LUISS) in Rome and has been hosted on its server. SMsocialnetwork.com and all its data were hosted on an Italian server and MySQL database managed by Aruba Group. The full Italian version of the survey is available in Multimedia Appendix 1.

### Measures and Procedures

The 7 main areas of interest on the survey were as follows:

1. Utility (5 items) measured how well the social network supports users in obtaining information, approaching and solving problems, making decisions, and attaining new insights about the disease. For each item, users gave a score on a Likert scale from 1 (not useful at all) to 7 (very useful).
2. Proximity (6 items) measured the user feeling of closeness with other social network users considering real-life relationships, dynamic chatting, and private messaging as well as interesting, supportive, or sympathetic comments on their own posts. Users gave a score on a Likert scale from 1 (do not agree at all) to 7 (strongly agree) (items 1 to 4) and on a Likert scale from 1 (not useful at all) to 7 (very useful) (items 5 and 6).
3. Sharing (6 items) measured how free the user felt to share private life information and general personal opinions in the social network activities (eg chat, posts, comments). Users gave a score on a Likert scale from 1 (do not agree at all) to 7 (strongly agree).
4. Interaction (8 items) measured the user ability to get in touch with other users in the community, playing an active role not only online but also in real life (eg, personal meeting, phone communications). Users gave a score on a Likert scale from 1 (do not agree at all) to 7 (strongly agree).
5. Solving uncertainty (4 items) measured the user’s opinion on innovative, effective, and practical solutions regarding MS-related health conditions and management. User gave a score on a Likert scale from 1 (none) to 5 (a lot).
6. Suggestion attitude (5 items) measured user attitude on playing an active or passive role in proposing new suggestions about innovative therapeutic approaches and treatment options. Users gave a score on a Likert scale from 1 (completely passive attitude) to 5 (very active role).
7. Exploration (5 items) measured user tendency to explore other websites related to MS (eg, Web communities, thematic pages on general social networks, blogs, forum, chats). User gave a score on a Likert scale from 1 (not explorative at all) to 7 (very explorative).
8. At the end of the survey, users were asked to complete a satisfaction rating on SMsocialnetwork.com based on a Likert scale from 1 (not satisfied at all) to 7 (very satisfied) including items concerning degree of satisfaction regarding personal experience in the SMsocialnetwork.com community and degree of complexity regarding personal experience in the SMsocialnetwork.com community.

### Statistical Analysis

Descriptive statistics are presented as relative frequencies, medians or means, and standard deviations, where applicable. Factorial analysis was performed to confirm the hypothesized domain structure and was implemented for each area of interest, considering only the first factor. Cronbach alpha was used to evaluate internal consistency reliability of each factor. Values above 0.70 for Cronbach alpha and above 0.8 for variance were considered acceptable. Items were excluded if they exhibited a low correlation with the construct, having a communality or item-rest correlation lower than 0.2. To evaluate consistency of items separately in each area of interest, we used factorial analysis to confirm that these items represent the same construct. Stata 13.0 (StataCorp LLC) was used for all analyses.

## Results

From April to June 2015, surveys from 202 users were collected from a total of 1020 active users. We excluded 72 questionnaires because of incomplete answers; 130 questionnaires (males 19/62, 30.6%, and females 43/62, 69.4%) were considered for the analysis. The response rate, defined as the percentage of users who filled out the survey over the total number of active users, was 12.74%; 1.6% (1/63) of users were younger than 20 years, 44.4% (28/63) were between ages 20 and 39 years, 34.9% (22/63) were between ages 40 and 54 years, 14.3% (9/63) were between ages 55 and 59 years, and 4.8% (3/63) were older than 60 years (see Table 1 for further data).

Utility (5 items) median values were 4 or 5 for all items (see Multimedia Appendix 2 for details). The factorial analysis confirmed the consistency of utility: first factor accounted for 100% of variance and Cronbach alpha coefficient was 0.95. All items had a high communality and item-rest correlation. The mean utility was 4.2 (SD 1.8) and the median (Q1-Q3) value was 4.2 (2.8-5.6).

**Table 1:** Demographic and clinical data.

Characteristic		Response rate (%)	Totals
**Gender, n (%)**		47.7	
	Female		43 (69.4)
	Male		19 (30.6)
**Age, n (%)**		48.5	
	<20 years		1 (1.6)
	20-39 years		28 (44.4)
	40-54 years		22 (34.9)
	55-59 years		9 (14.3)
	>60 years		3 (4.8)
**Education, n (%)**		49.2	
	8 years		9 (14.1)
	13 years (high school)		24 (37.5)
	3-year degree		10 (15.6)
	Master’s degree		15 (23.4)
	PhD		3 (4.7)
	Other		3 (4.7)
Disease duration, years, mean (SD)		90.0	7.7 (7.5)
Treatment/therapy duration, years, mean (SD)		86.9	6.7 (6.9)
**Frequency of SMsocialnetwork.com access, n (%)**		95.4	
	≤1 time per month		43 (34.7)
	1 time per week		18 (14.5)
	>1 time per week		40 (32.3)
	Daily		16 (12.9)
	>1 time per day		7 (5.7)
**Frequency of SMsocialnetwork.com access compared to total Internet access, n (%)**		93.8	
	All the time		15 (12.3)
	>1 time		33 (27.1)
	Sometimes		32 (26.2)
	Few times		19 (15.6)
	Rarely		23 (18.9)
**Self-reported health status, n (%)**		96.2	
	Excellent		10 (8.0)
	Good		35 (28.0)
	Average		44 (35.2)
	Not very good		29 (23.2)
	I prefer not to answer		5 (4.0)
	Other		2 (1.6)

**Table 2:** Analysis results summary of the areas of interest.

	Mean (SD)	Median	Q1-Q3	%	Maximum	Alpha
Utility	4.2 (1.8)	4.2	2.8-5.6	60	7	0.95
Proximity	4.5 (1.3)	5	3.7-5.5	71	7	0.85
Sharing	5.3 (1.4)	5.6	4.4-6.2	80	7	0.85
Interaction	3.9 (1.5)	4.1	2.9-5.3	59	7	0.9
Solving uncertainty	2.8 (0.8)	3	2-4	60	5	0.79
Suggestion attitude	3.3 (1.0)	3	3-4	60	5	0.76
Explore	4.7 (1.3)	5.3	4.6	76	7	0.85

Proximity (6 items) median values were 4 or 5 for all items (see Multimedia Appendix 2). The factorial analysis confirmed the consistency of proximity: first factor accounted for 82% of variance and Cronbach alpha coefficient was 0.85. All items had a high communality and item-rest correlation. The mean proximity was 4.5 (SD 1.3) and the median (Q1-Q3) value was 5 (3.7-5.5) (see Table 2).

Sharing (6 items) median values were 5 or 6 for all items except for item “I use a nickname to distinguish myself in this community” (see Multimedia Appendix 2) that also showed a poor communality (0.001) and item-rest correlation (0.05) and was not included in the final analysis. The factorial analysis confirmed the consistency of sharing: first factor on the residual 5 items accounted for 100% of variance and the Cronbach alpha coefficient was 0.85. All residual items had a high communality and item-rest correlation. The mean sharing was 5.2 (SD 1.4) and the median (Q1-Q3) value was 5.6 (4.4-6.2) (see Table 2).

Interaction (8 items) median values ranged from 3 to 6 (see Multimedia Appendix 2). The factorial analysis confirmed the consistency of interaction: first factor accounted for 80% of variance and Cronbach alpha coefficient was 0.90. All items had a high communality and item-rest correlation. The mean interaction was 3.9 (SD 1.5) and the median (Q1-Q3) value was 4.1 (2.9-5.3) (see Table 2).

Solving uncertainty (4 items) median value was 3 for all items (see Multimedia Appendix 2). The factorial analysis confirmed the consistency of solving uncertainty: first factor accounted for 100% of variance and Cronbach alpha coefficient was 0.79. All items had a high communality and item-rest correlation. The mean solving uncertainty was 2.8 (SD 0.8) and the median (Q1-Q3) value was 3 (2-4) (see Table 2).

Suggestion attitude (5 items) median values ranged from 2 to 4 (see Multimedia Appendix 2). The meaning of the Likert scale in the item “In the discussions on treatments and drugs, you are more prone to listen/convince the other members of the contrary” (ie, high value meant low attitude to suggest and low value showed a high attitude to suggest) with respect to the other item scales and the relative values was reversed in the factorial analysis. This item and the item “In the discussion on treatment and drugs, you are more prone to listen or talk” showed a poor communality and item-rest correlation (0.0 and 0.17), and they were not included in the final analysis. The factorial analysis confirmed the consistency of suggestion attitude: first factor on the residual 3 items accounted for 100% of variance and the Cronbach alpha coefficient was 0.76. The residual 3 items had a high communality and item-rest correlation. The mean suggestion attitude was 3.3 (SD 1.0 and the median (Q1-Q3) value was 3 (3-4) (see Table 2).

Exploration (5 items) median values ranged from 3 to 6 (see Multimedia Appendix 2). The meaning of the Likert scale of the item “Usually I am not interested in visiting new websites” was contrary (ie, high value meant low attitude to explore and low value showed a high attitude to explore) with respect to the other item scales and the relative values were reversed. This item was removed because it showed a low communality and a low item-rest correlation. The factorial analysis confirmed the consistency of exploration: on the residual 4 items, Cronbach alpha coefficient was 0.85. The residual 4 items had a high communality and item-rest correlation. The mean explore was 4.7 (SD 1.3) and the median (Q1-Q3) value was 5.3 (4-6) (see Table 2).

Only solving uncertainty had low alpha (>0.8) and communality (0.2) scores. However, we considered the values ≥0.8 for variance and ≤0.2 for communality or item-rest correlation acceptable.

**Figure 1:**
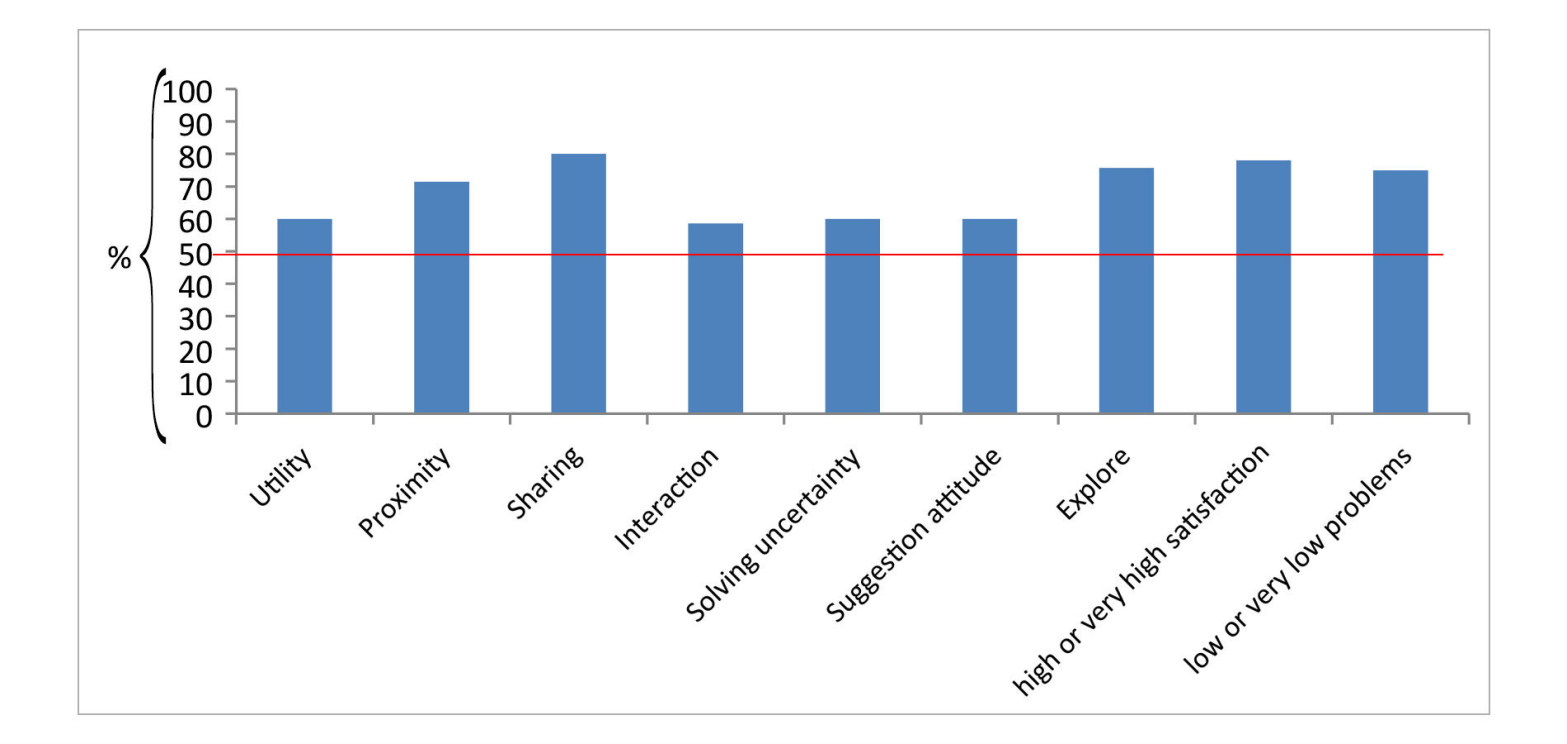
Median scores of survey main areas and degree of satisfaction regarding personal and problematic experiences in SMsocialnetwork.com, reported as a percentage of the relative Likert scales (from 1 to 5 or 7).

For each area of interest, the range and meaning of the Likert scale corresponded to the same range and meaning of each item used to identify it (ie, low value equals low degree of agreement and high value equals high degree of agreement). Finally, 78.2% of SMsocialnetwork.com users showed a high or very high degree of satisfaction regarding personal experience in the SMsocialnetwork.com community, and 75% of SMsocialnetwork.com users showed a low or very low degree of personal problematic experiences in the SMsocialnetwork.com community (see Figure 1).

## Discussion

### Principal Findings

In our analysis, the median scores of the areas of interest were above (utility, proximity, sharing, interaction, and exploration) or equal (solving uncertainty and suggestion attitude) to the intermediate value of the Likert scale. These data indicated, in line with the previous work of Oxman and Guyatt [37], a satisfaction with SMsocialnetwork.com (see Figure 1). Indeed, users positively evaluated SMsocialnetwork.com in the following areas:

1. Obtaining information, improving the approach to solve problems and making decisions, and providing new insights about MS
2. Improving the feeling of closeness toward other social network users
3. Catalyzing relationships, sharing private life information, and texting general personal opinions
4. Getting in touch with other users playing an active role especially online but also, to a lesser extent, in real life
5. Receiving innovative, effective, and practical solutions regarding MS-related issues and management
6. Delivering information about innovative treatment options

Furthermore, SMsocialnetwork.com users showed a tendency to explore other Web pages related to MS (eg, Web communities, thematic pages on general social networks, blogs, forum, chats) and exhibited both a high degree of satisfaction and a low degree of problematic experiences in the SMsocialnetwork.com community.

Social networking is one of the major players in the current era of transformational changes in how information is accessed and shared [38]. It is known that social networks transmit media such as video, blogs, ratings and reviews, podcasts, and audio among a group of people who are linked by a common characteristic, such as likes and dislikes [39]. Although social networks are considered primarily a recreational tool, they are becoming increasingly important to businesses and organizations [40]. Specifically, social networks hold considerable potential value for health care organizations because they can be used to reach stakeholders, aggregate information, and leverage collaboration [28,41]. In health-related areas, social networks users may find a way to track progress about the disease and access disease information, learn from real-world experiences of other people with the same medical condition, share their findings with other patients and with health care professional organizations, and create a virtual space where patients and caregivers give and receive support [42-45]. The high number of disease-related Internet pages is likely due to the ease of taking advantage of the Web’s opportunities through the virtual environment. Recent research showed that a high number of Americans get information about therapies or diseases online [5] and a very high number of physicians and nurses are interested in using social networks for professional purposes. Because both patients and clinicians are using social networks, health care organizations have an opportunity to leverage multiple audiences. Recently, it has been recorded that more than 700 of the 5000 US hospitals count on social media and social networking to enhance their ability to communicate to stakeholders. Despite a high number of Web pages dedicated to MS, to the best of our knowledge, there are no social networks specifically dedicated to MS, and SMsocialnetwork.com is the first social network to share evidence-based information with the added value of the constant online presence of the medical experts. Using a Web-based survey, we investigated SMsocialnetwork.com as a shoppable condition [12,13,20] in which users may acquire information to support health-related coping and their social interaction.

We observed that MS patients interact on the Internet not only about MS-related issues but also on general personal opinions and private life information (sharing). Moreover, users showed a tendency to play an active role online by dynamic chatting and private messaging as well as posting interesting, supportive, or sympathetic comments (proximity). We observed a low user tendency to get in touch with other users in real life (interaction). While SMsocialnetwork.com users appreciate an intimate, close, and empathic community, they prefer an online relationship than face-to-face interaction compared to people in nonthematic Web communities. Indeed, the users of generalist social networks (eg, Facebook) seem to have close relationships in real life, and only a small percentage of social network users have never met or only met their Internet-based friends once [46], supporting that online social media are not a substitute for real-life interpersonal exchanges but offer a different experience that brings people together. We cannot exclude the role of MS disability on the reduced user real-life interpersonal relationships, as previously found in other chronic diseases [47-50].

### Limitations

In this study, we collected 130 questionnaires, which may be considered a small number for a factorial analysis. However, we used the factorial analysis only to confirm the construct identified by items; on these premises our results should not have been affected by a larger sample size. Moreover, our response rate is in accordance with the mean response rate of social network–based studies, ranging from 2% to 27% with an average of 12%, generally considered as an indicator of reliable data quality for social network–based studies [51,52].

We excluded 35.6% of the surveys from the final analysis, which had more than 20% of the missing data. This is in line with the very high percentage of missing data generally observed in online surveys, where the anonymity allows inaccurate answers or early interruption of the questionnaire (missing data range from 15% to 20% in quantitative research) [53].

We cannot compare our data with those from other MS social networks without constant online presence of the medical team. Indeed, to the best of our knowledge, there are no social networks specifically dedicated to MS.

In our survey there were no items directly investigating the appreciation of MS experts’ contribution in SMsocialnetwork.com. However, in our factorial analysis, median scores of the areas of interest indicated, in any case, a general satisfaction [37] with the Web platform (see Figure 1). Obviously, we can only speculate about the advantage brought by the constant online presence of the SMsocialnetwork.com medical team. Finally, SMsocialnetwork.com did not provide an explanatory tutorial that would have been useful to facilitate users completing the survey [52].

### Conclusion

In this study, we observed that an MS-dedicated social network (SMsocialnetwork.com) is perceived by users as a useful tool to receive information and solve problems in daily life, providing innovative and effective insights about MS health care. Moreover, users were prone not only to give information but also to hear and listen to others, and they did not try to convince and impose their opinions; they appeared to like discussing personal problems at the same level with others. We speculate that users were reassured by SMsocialnetwork.com experts’ constant online presence and competence. We also believe that an MS-dedicated social network may allow MS experts to reach a deeper comprehension of the needs of people with MS and may suggest how to improve both medical communications and clinical empathy, likely configuring a new kind of therapeutic alliance between physicians and patients.

## Appendix

Multimedia Appendix 1 Survey questionnaire in original language (Italian).

Multimedia Appendix 2 Survey items.

## Abbreviations

LUISS: Free International University for Social Studies “Guido Carli”
MS: multiple sclerosis
