# Supplementary material for: Health-Related Coping and Social Interaction in People with Multiple Sclerosis Supported by a Social Network: Pilot Study With a New Methodological Approach
Source: Interact J Med Res. 2017 Jul 14;6(2):e10. doi: 10.2196/ijmr.7402 (PMC5533941; doi:10.2196/ijmr.7402)
Supplement: Multimedia Appendix 1 [file ijmr_v6i2e10_app1.pdf]

Data entry

Innovazione e Social media

	**Datestamp:**	
	**IP address:**	NULL

Le domande sul Sito smsocialnetwork

G1_Q0001	**Da quanto tempo stai affrontando la Sclerosi Multipla?**	Anni<br>Mesi
G1_Q0002	**Da quanto tempo sei in cura con trattamenti medici ed utilizzo di medicinali?**	Anni<br>Mesi
G1_Q0003	**Quante volte accedi al smsocialnetwork?**	Please choose..
G1_Q0004	**In quale misura accedi a smsocialnetwork rispetto a quante volte accedi a internet?**	Please choose..
G1_Q0005	**Attualmente come giudichi il tuo stato di salute?**	Please choose..

PRESENZA IN INTERNET

G2_Q0001	**Indica in quale misura smsocialnetwork ti ha aiutato per: Scegli la risposta più appropriata per ogni affermazione 1(nulla) e 7 (moltissimo)**	ricevere informazioni<br>imparare come fare delle cose<br>avere nuove idee<br>risolvere problemi

		intraprendere decisioni	
G2_Q0002	**Indica in quale misura sei d’accordo o non sei d’accordo con le seguenti citazioni.**	uso frequentemente chat istantanea per parlare con gli altri membri di questa comunità	
		in genere conosco gli altri utenti connessi nel sito	
		Gli altri utenti rispondono velocemente ai miei post	
		Gli altri utenti rispondono velocemente ai miei messaggi privati	
G2_Q0003	**Quando discuti con i tuoi amici all’interno del social network su medicinali e trattamenti, tu:**		Please choose..
G2_Q0004	**Indica in quale misura sei d’accordo con le seguenti**	racconto la mia storia agli altri membri della	

	**affermazioni.**	comunità	
		condivido foto o altre informazioni personali con gli altri membri di questa comunità	
		esprimo le mie opinioni nei miei post	
		mostro le mie reali informazioni personali nel mio profilo	
		uso uno pseudonimo in questa comunità che mi differenzia dagli altri membri	
G2_Q0005	**Indica il tuo livello di soddisfazione cliccando nella risposta a destra**	Tutto sommato, sono soddisfatto della mia esperienza in questa community	
		Generalmente, mi fa piacere interagire con altre persone in questa community	
G2_Q0006	**Con che frequenza i tuoi amici nella della community ti chiedono di trattamenti e medicinali?**	Please choose..	

PRESENZA IN INTERNET 2

G3_Q0001	**Indica in quale misura sei d’accordo o non sei d’accordo con le seguenti dichiarazioni:**	in quale misura hai la sensazione di “essere molto vicino agli altri” ?	
		in quale misura hai la sensazione di essere insieme con gli altri ?	
G3_Q0002	**Indica quanto sei d’accordo cliccando nelle risposte a destra**	spesso aiuto gli altri membri di questa community che hanno bisogno di informazioni o aiuto	
		ho un ruolo attivo in questa community	
		ho dato consigli in questa community	
		i miei consigli sono stati applicati dagli altri membri di questa community in maniera positiva	
G3_Q0003	**Come descrivi le tue attività offline? Rispondi alle seguenti affermazioni**	contatto telefonicamente i membri di questa community	

incontro informalmente i membri di questa community

partecipo attivamente ai regolari incontri organizzati al di fuori della community

partecipo alle attività organizzate dalla community online

G3_Q0004	**nelle discussioni su medicinali e trattamenti, tu sei più propenso:**	Please choose..
G3_Q0005	**nelle discussioni su medicinali e trattamenti cosa ti capita maggiormente?**	Please choose..
G3_Q0006	**In genere, quando discuti con altri membri del tuo gruppo, tu sei:**	Please choose..
G3_Q0007	**si prega di dare il nome di tre persone che ti permettono sempre di ottenere qualche consiglio**	1)<br>2)<br>3)
G3_Q0008	**Indica il nome di persone che in passato ti hanno dato informazioni utili su medicinali e trattementi:**	1)<br>2)<br>3)<br>4)

		5)	

INNOVAZIONE PERSONALE

G4_Q0001	**Indica in quale misura sei d’accordo o non sei d’accordo con le seguenti affermazioni**	mi piace esplorare altri siti web	
		quando so di un nuovo sito web trovo sempre una scusa per visitarlo	
		tra gli i utenti della community io sono di solito il primo a visitare nuovi siti	
		in generale non sono molto interessato nel visitare nuovi siti	
		quando ho tempo libero, di solito visito nuovi siti web	

INNOVAZIONE

G5_Q0001	**In quale misura hai trovato problemi nella community?**	Please choose..
G5_Q0002	**In quale misura hai trovato soluzioni nella nuova community?**	Please choose..

EFFETTIVITA'

G6_Q0001	**Quanto sono effettive le soluzioni proposte**	Please choose..

	**in questo sito web per la tua situazione?**	
G6_Q0002	**Quanto effettive sono le nuove idee proposte nel sito web per il tuo stato di salute?**	Please choose..
G6_Q0003	**Quante sono le nuove pratiche suggerite dagli altri in merito agli effetti collaterali?**	Please choose..
	**Adozione**	
G7_Q0001	**Hai mai applicato il suggerimento trovato in un forum?**	Please choose..
G7_Q0002	**Quanto hai seguito precisamente il consiglio suggerito?**	Please choose..
G7_Q0003	**In quale misura il contenuto di un messaggio ti ha motivato ad intraprendere un'azione?**	Please choose..
G7_Q0004	**In quale misura sei d'accordo con le azioni suggerite nel messaggio?**	Please choose..
	**Demografia**	
Q40	**Quale è il tuo username nella community? (opzionale ma raccomandato)**	
G8_Q0001	**Quanti anni hai?**	Please choose..
G8_Q0002	**sesso:**	Please choose..
G8_Q0003	**qual è il tuo livello più alto di educazione?**	Please choose..
